# Epidemiology and microbiology of Gram-positive bloodstream infections in a tertiary-care hospital in Beijing, China: a 6-year retrospective study

**DOI:** 10.1186/s13756-018-0398-x

**Published:** 2018-09-03

**Authors:** Qiang Zhu, Yan Yue, Lichen Zhu, Jiewei Cui, Minghui Zhu, Liangan Chen, Zhen Yang, Zhixin Liang

**Affiliations:** 1 Department of Respiratory Medicine, Chinese PLA General Hospital, Fuxing Road No. 28, Beijing, 100853 China; 2 Department of Respiratory Medicine, Affiliated Hospital of Nantong Third People’s Hospital, Qingnian Central Street No. 99, Jiangsu Province, 226000 China; 3 The postgraduate department, Chinese PLA General Hospital, Beijing, 100853 China

**Keywords:** Gram-positive bacteria, Bloodstream infections, Epidemiology, Incidence, Resistance, Outcome

## Abstract

**Background:**

Gram-positive bacterial bloodstream infections (BSIs) are serious diseases associated with high morbidity and mortality. The following study examines the incidence, clinical characteristics and microbiological features, drug resistance situations and mortality associated with Gram-positive BSIs at a large Chinese tertiary-care hospital in Beijing, China.

**Methods:**

A retrospective cohort study of patients with Gram-positive BSIs was performed between January 1, 2011, and June 31, 2017, at the Chinese People^’^s Liberation Army General Hospital. The patients’ data were collected and included in the reviewing electronic medical records.

**Results:**

A total of 6887 episodes of Gram-positive BSIs occurred among 4275 patients over 6 years, and there were 3438 significant BSI episodes 69% of these cases were healthcare-associated, while 31% were community-associated. The overall incidence of Gram-positive BSIs fluctuated from 7.26 to 4.63 episodes per 1000 admissions over 6 years. Malignancy was the most common comorbidity and indwelling central intravenous catheter was the most common predisposing factor for BSI. *Staphylococci* were the major pathogen (65.5%), followed by *Enterococcus spp:*(17.5%), *Streptococcus spp.*(7.1%) and other bacterial pathogens (9.9%). The resistance rates of *Staphylococci* and *E.faecium* to penicillins were more than 90%. the vancomycin-resistant isolates were *E. faecium* (4.1%) and *staphylococcus epidermidis* (0.13%); and only *E.faecalis* and *E.faecium* showed resistance to linezolid (3.8% and 3.1%). Between 2011 and 2017, the overall mortality of Gram-positive BSIs decreased from 6.27 to 4.75% (X^2^ = 0.912, *p* = 0.892). Neverthess, the mortality in the ICU decreased from 60.46 to 47.82%, while in the general ward it increased from 39.54 to 52.18%.

**Conclusions:**

The morbidity and mortality of Gram-positive BSIs have showed downward trends. Vancomycin and linezolid are still consider the best treatment for patients with Gram-positive BSIs.

## Background

BSIs are serious diseases associated with high morbidity and mortality, which are diffcult to treat and ofen result in a heavy social and economic burden [1]. In China, BSIs account for one-third of healthcare-associated infection, followed by lower respiratory tract and urinary tract infections [2]. Acoording to a resent USA_based prevalence surey, Gram-positive pathogens were the most frequently isolated pathogens among BSIs [3]. Acoording to CHINET, the most commonly gram-positive in 2010 were *Staphylococci*, *Enterococcus spp.* and *Streptococcus spp.* [4, 5]. In addition, some studies have reported that the crude incidence of BSIs varies from country to country [6–8].

Over recent years, due to the widespread use of antibiotics, immunosuppressants and anti-tumour drugs, and the increase of invasive medical examinations and treatments, the epidemiology and antimicrobial resistance have been changing. Multidrug resistant patterns in Gram-positive bacteria have resulted in difficult-to-treat or even untreatable cases, which in tum caused the increase in mortality. E.g. recent ECDC data from 2013 indicated a global increase in methicillin-resistant *staphylococcus aureus* (MRSA) (> 50%) isolated from blood, and a higher rate in vancomycin- resistant Gram-positive coccus in Europe [9–11]. Morever, *Enterococcus spp.* has shown increasing prevalence of acquired resistance to penicillins, aminoglycosides and vancomycin, which was observed in many countries [12, 13]. A report from Europe has shown that there were more than 1.2 million episodes of BSIs with 157,000 deaths per year, while between 57,500 and 677,000 episodes of BSIs were reported in North America causing some 79,000 to 94,000 deaths [14].

Although there are number of study on BSIs, few data reports have focus on Gram-positive bacteria- BSIs and the epidemiology and antimicrobial resistance of various BSIs in different periods and regions, thus making it very difficult to timely choose antibiotic treatment based on the empirical evidence. Therefore, the purpose of this study was to provide more data on the incidence, microbiological features, mortality and drug resistance data at our hospital.

## Methods

We performed a retrospective cohort study in patients with Gram-positive bacterial bloodstream infections between January 1, 2011, and June 31, 2017 at the Chinese People’s Liberation Army General Hospital (PLAGH), a 2200-bed tertiary-level healthcare facility in Beijing, China.

Eligible patients included all patients with at least one positive blood culture for Gram-positive bacteria. In patients with persistent BSIs caused by the same organism, only the first episode was included within the previous 30 days. If patients had 2 or more separate BSIs, each infection was considered individually. All patients were identified by searching the real-time nosocomial infection surveillance system (RT-NISS) [15]. This platform utilizes data from electronic medical record systems, such as sex, hospital ward, comorbidity, and microbiology results, with the application of clinically validated algorithms to identify and classify all of the patients’ infections. The infection management and disease control department of PLAGH developed RT-NISS. In addition, since Gram-positive bacilli are not routinely identified at our hospital, patients with those types of bacteria were excluded them from the study.

### Data collection

The patients’ data were collected and included in the reviewing electronic medical records. We recorded the demographic data, including age and sex; the clinical data included predisposing factors, the hospitalization wards, and comorbidities. The microbiological data included species of Gram-positive bacteria, likely sources of BSIs (identified by treating doctors and/or physicians of the infection management and disease control department), and antimicrobial susceptibility results. We aslo collected annual admission data to calculate incidence rates, which are expressed as the number of BSI episodes per 10,00 hospital admissions.

### Definitions

The diagnosis of Gram-positive bacterial BSIs had to meet the following criteria: 1) isolation of Gram-positive bacteria from one or more blood cultures; 2) having one of the following symptoms: fever (> 38 °C), chills, or hypotension; and 3) elimination of the possibility of contamination during the collection and cultivation of blood samples [16]. Healthcare-associated BSIs included episodes of bacteraemia occurring more than 48 h after admission and < 7 days after discharge in patients undergoing intensive outpatient therapy involving regular hospital contact. Healthcare-associated Gram-positive BSIs were defined as the first positive blood culture obtained ≥48 h after hospital admission and with no evidence of infection at admission [16]. An episode was defined as the positive isolation of the Gram-positive bacteria from at least one blood culture sample from a patient and without a prior blood culture isolating the same bacteria within the previous 30 days [6]. Onset of BSIs was defined as the date when the blood culture was collected. Poly-microbial BSIs were defined as two or more clinically important organisms isolated from one single blood culture sample or different blood culture samples within 48 h. Active agents were confirmed according to the antibiotic susceptibility testing.

### Identification and antibiotic susceptibility testing

Blood was cultured using a BacT/ALERT 3D system (Becton-Dickinson, Sparks, MD, USA) in the microbiology laboratory. Species identification was performed using the VITEK 2 system (BioMérieux, Marcy l′Etoile, France). Antibiotic susceptibility testing was performed using the VITEK 2 system or the Kirby-Bauer Disk Diffusion method (Oxoid, UK) according to the recommendations proposed by the Clinical and Laboratory Standards Institute (CLSI) [17].

### Statistical analysis

Categorical variables are expressed as frequency counts and percentages with 95% confidence intervals (95% CIs). Continuous variables are expressed as medians and inter-quartile ranges. The incidence and mortality of Gram-positive BSIs over these years were determined using chi-square test for trend. The comparison of categorical variables was performed using Pearson’s chi-square test or Fisher^’^s exact test, while the comparison of continuous variables was performed using the Mann-Whitney U test. The results with a 2-tailed *p*-value < 0.05 were considered statistically significant. All of the statistical analyses were performed using SPSS software, version 20.0 (IBM Corp, Armonk, NY, USA).

## Results

### Incidence and species distribution

In total, 6887 episodes of BSIs caused by Gram-positive bacteria occurred among 4275 patients during the 6-year study period. Among 4275 patients with Gram-positive BSIs, 527 were infectioned with two different species (> 30 days apart), and 881 had 2 or more BSI with the same species < 30 days apart. Only the results for the first cases among repeat results were included, so there were 3438 significant BSI episodes.

Among all BSI isolates, the most common Gram-positive bacterial species was *Staphylococcui* (65.5%), followed by *Enterococcus spp.* (17.5%), *Streptococcus spp.*(7.1%) and other bacterial pathogens (9.9%). The species proportions in each year are shown in Table 1**.** In addition, 69% of these cases were healthcare-associated infections, 31% were community acquired, and *Staphylococci* was the most common isolate. For all Gram-positive BSIs species, healthcare-associated infection was more common than community-acquired infection except for *Streptococcus.*

**Table 1:** The species ratio from 2011 to 2017(6-months’ data)

organisms	year							
	2011(926)	2012(1102)	2013(897)	2014(970)	2015(1145)	2016(1338)	2017(509)	total(6887)
staphylococcus	608^a^(65.7)^b^	697(63.3)	580(64.7)	632(65.2)	751(65.6)	937(70.1)	303(59.5)	4508(65.5)
enterococcus	182(19.7)	208(18.9)	136(15.2)	151(15.5)	202(17.6)	217(16.2)	114(22.4)	1210(17.5)
streptococcus	59(6.3)	87(7.9)	89(9.9)	67(6.9)	75(6.6)	66(4.9)	42(8.3)	485(7.1)
others	77(8.3)	110(9.9)	92(10.2)	120(12.4)	117(10.2)	118(8.8)	50(9.8)	684(9.9)

^a^: The bolded data indicates the total number

^b^:The percentage of that group

**Fig. 1:**
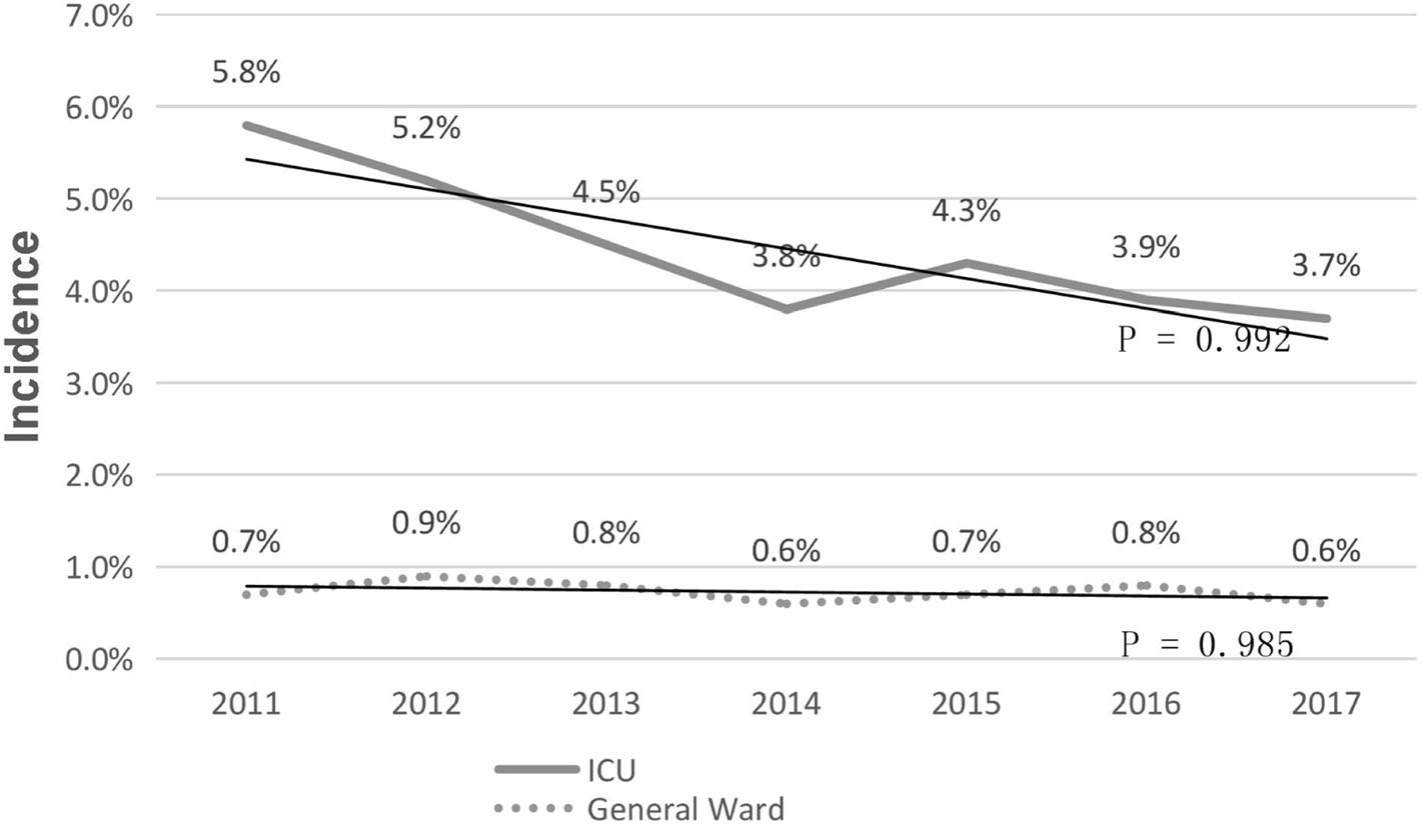
Incidences of BSIs due to ICU and General ward from 2011 to 2017(6-months’ data)

The overall incidence of Gram-positive BSIs was 6.94 episodes per 10,00 admissions, and the rate decreased from 7.26 to 4.63 episodes per 10,00 admissions(X^2^ = 1.0, *p* = 0.986) over 6 years (7.26 in 2011, 7.83 in 2012, 6.57 in 2013, 6.41 in 2014, 7.21 in 2015, 7.95 in 2016, 4.63 in 2017). Respectively, the overall incidence in each year showed a downward trend in the intensive care unit (X^2^ = 0.812, *p* = 0.992), while there was no obvious change in the general ward(X^2^ = 1.02, *p* = 0.985) (Fig. 1).

### Demographic and clinical characteristics

A total of 6887 episodes of Gram-positive BSIs occurred among 4275 patients over the 6 years. Demographics and clinical data were available for 1330 of these clinically relevent episodes, as shown in Table 2. The median age was 53 years old (95% CI 51–55%), and 870 (65%, 95% CI 48–81%) of patients were male. The age distribution had a certain relationship with the distribution of strains (*p* < 0.001), with *Enterococcal* BSIs being associated with older age. Malignancy was the most common comorbidity (28%), followed by hypertension (15%). Moreover, The Gram-positive BSIs incidence was 11.5 patients per 10,00 in the medical ward every year, 9.5 patients per 10,00 in the surgical ward, and 63.3 patients per 10,00 in the ICU, and the distribution of these clinically-relevant episodes in surgical ward were statistically different from our study (*p* < 0.05). Central intravenous catheters were the most common predisposing factors (11%), followed by chemoradiotherapy (6%) and indwelling urinary catheters (4%).

**Table 2:** Demographic and clinical characteristics of patients with BSIs

	Staphylococcus aureus(*n* = 227)	CoNS(*N* = 446)	enterococcus(*n* = 499)	Viri-dans streptococcus(*n* = 148)	Streptococcus pneumonia(*n* = 10)	total(*n* = 1330)	*p*-value
age	51(50–57)	53(51–55)	59(58–61)	52(50–54)	51(42–53)	53(51–55)	*p* < 0.001
Male	152(66,64–69)	284(64,58–69)	320(64,60–67)	108(73,71–74)	6(60,59–60)	870(65,48–81)	0.857
Female	75(34,30–35)	162(36,30–41)	179(36,32–39)	40(27,25–28)	4(40,39–40)	460(35,18–51)	0.455
Comorbidities							
Malignancy	68(30,27–32)	83(19,13–24)	175(35,31–38)	38(26,24–27)	6(60,59–60)	370(28,11–44)	*p* < 0.001
Trauma	15(7,4–9)	18(4,0–9)	20(4,0–7)	3(2,0–3)	1(10,9–10)	57(4,0–20)	0.408
Hypertension	35(15,12–17)	74(17,11–22)	69(14,10–17)	23(16,14–17)	2(20,19–20)	203(15,0–31)	0.994
Heart disease	15(7,4–9)	69(15,9–20)	38(8,4–11)	10(7,5–8)	1(10,9–10)	133(10,0–26)	0.007
Diabetes mellitus	25(11,8–13)	45(10,4–15)	54(11,7–14)	9(6,4–7)	3(30,29–30)	136(10,0–26)	0.007
Hematological disease	21(9,6–11)	12(3,0–8)	15(3,0–6)	8(5,3–6)	1(10,9–10)	57(4,0–20)	0.006
Hospital Ward							
Medical	117(52,49–54)	315(71,65–76)	305(61,56–63)	97(66,64–67)	10(100,99–100)	844(63,46–79)	0.219
Surgical	110(48,45–50)	131(29,23–34)	194(39,35–42)	51(34,32–35)	0(0,0–0)	486(37,20–53)	0.003
ICU	50(22,19–24)	122(27,21–32)	132(26,22–29)	28(19,17–20)	0(0,0–0)	332(25,9–41)	0.145
Predisposing factors							
central intravenous catheter	62(27,24–29)	42(9,3–14)	37(38,37–39)	4(3,1–4)	1(10,9–10)	146(11,0–27)	*p* < 0.001
indwelling urinary catheter	26(11,8–130	11(2,0–7)	18(18,17–19)	1(1,0–2)	0(0,0–0)	56(4,0–20)	*p* < 0.001
Immunosuppressive	22(10,7–12)	8(2,0–7)	8(8,7–9)	3(2,0–3)	2(20,19–20)	43(3,0–19)	*p* < 0.001
chemoradiotherapy	41(18,15–20)	21(5,0–10)	15(15,14–16)	4(3,1–4)	1(10,9–10)	82(6,0–22)	*p* < 0.001
endotracheal intubation	12(5,2–7)	11(2,0–7)	14(14,13–15)	1(1,0–2)	0(0,0–0)	38(3,0–19)	0.195
tracheostomy tube	6(3,0–5)	3(1,0–6)	6(6,5–7)	0(0,0–1)	0(0,0–0)	15(1,0–17)	0.211
Significant isolates							
Nosocomial	164(72,69–74)	312(70,64–75)	400(80,72–85)	50(34,32–35)	5(50,49–50)	931(70,53–86)	*p* < 0.001
Community acquired	63(28,25–30)	134(30,24–35)	99(20,31–38)	98(66,64–67)	5(50,49–50)	399(30,13–46)	*p* < 0.001

### Antimicrobial susceptibility and outcomes

Antimicrobial resistance levels for the most common organisms of the Gram-positive BSIs are shown in Table 3. *Staphylococci* showed a high level of resistance to penicillin, ampicillin, erythromycin, and ciprofloxacin; the resistance of MRSA to penicillin and ampicillin was 100%, and the resistance of MSSA to penicillin was 96.2% but to ampicillin was 40.9%; *Staphylococci* was susceptible to gentamicin and tetracycline except for MRSA and *Staphylococcus haemolyticus*; only *Staphylococcus epidermidis* showed resistance to linezolid at a rate of 0.13% (1/756); and no vancomycin resistance was found. *E. faecalis* showed great sensitivity to penicillin, ampicillin, gentamicin, erythromycin, and ciprofloxacin, but *E. faecium* showed the opposite antibiotic resistance. The rates of Linezolid resistance of *E. faecalis* and *E.faecium* were 3.8% (5/132) and 3.1% (9/294), respectively, but only *E.faecium* showed resistance to vancomycin at a rate of 4.1% (12/294). There was no linezolid resistance or vancomycin resistance with *Streptococcus spp. viridians streptococci* that showed no resistance to penicillin, ampicillin, or clindamycin, but the rate of erythromycin resistance was 70.4%. However, *streptococcus pneumoniae* had a higher resistance rate than *viridians streptococci* in most of the antibiotics. In addition, since there is no national standard of the National Committee for Clinical Laboratory (NCCLS), it was not possible to judge the drug susceptibility of Gram-positive bacilli and others in the present study.

**Table 3:** Rates of antimicrobial resistance among gram-positive bacteria most frequently isolated from patients with BSIs

Microbiology	Antimicrobial drug n_ri_/n_rt_(%resistant)								
	Penicillin	Ampicillin	Gentamicin	Erythomycin	Ciprofloxacin	Clindamycin	Linezolid	Vancomycin	Tetracycline
staphylococcus									
Staphylococcus aureus	96.2%(153/159)	40.9%(65/159)	32.7%(52/159)	57.9%(92/159)	34.6%(55/159)	51.6%(82/159)	–	–	30.8%(32/104)
CNS	96.4%(429/445)	74.4%(331/445)	20.4%(91/445)	85.6%(381/445)	47.0%(209/445)	69.2%(308/445	–	–	*
staphylococcus epidermidis	96.7%(731/756)	88.0%(665/756)	10.2%(77/756)	85.1%(643/756)	56.1%(424/756)	45.2%(342/756)	–	0.13%(1/756)	2.2%(15/685)
MRSA	100%(68/68)	100%(68/68)	47.1%(32/68)	75.0%(51/68)	86.8%(59/68)	75.0%(51/68)	–	–	38.2%(26/68)
Staphylococcus hominis	91.0%(832/914)	74.4%(680/914)	2.7%(24/914)	91.2%(834/914)	52.6%(481/914)	68.3%(624/914)	–	–	42.8%(345/806)
Staphylococcus haemolyticus	96.0%(117/122)	94.3%(115/122)	72.1%(88/122)	95.1%(116/122)	84.4%(103/122)	52.5%(64/122)	–	–	32.0%(39/122)
enterococcus									
E.faecalis	9.1%(12/132)	11.4%(15/132)	31.1%(41/132)	53.4%(31/58)	35.6%(47/132)	*	3.8%(5/132)	–	59.5%(47/79)
E.faecium	92.5%(272/294)	91.5%(269/294)	70.4%(207/294)	74.3%(139/187)	90.1%(265/294)	*	3.1%(9/294)	4.1%(12/294)	43.8%(89/203)
Streptococcus									
Viri-dans streptococcus	0%(0/32)	0%(0/48)	*	70.4%(23/24)	*	0%(0/24)	0%(0/25)	0%(0/25)	*
Streptococcus pneumonia	30%(3/10)	80%(8/10)	*	90%(9/10)	0%(0/3)	100%(1/1)	0%(0/10)	0%(0/10)	*

*n*_*ri*_ number of resistant isolates, *n*_*rt*_ number of isolates tested

-: without resistant; *: without test

**Fig. 2:**
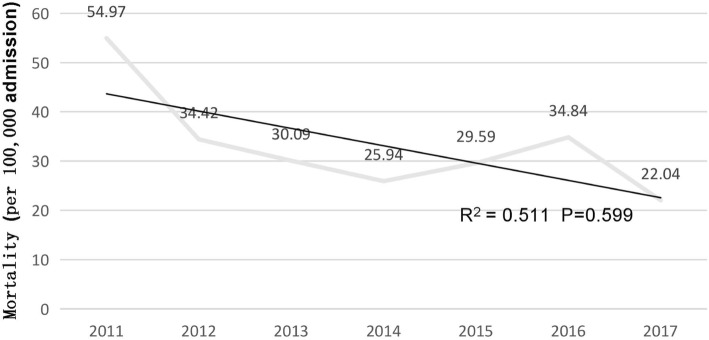
Mortality of the Gram-positive bacterials BSIs from 2011 to 2017(6-months’ data). The mortality from Gram-positive BSIs shows downward trend; the rate fluctuated from 54.97 to 22.04 episodes per 100,000 admissions during the 6 years

**Fig. 3:**
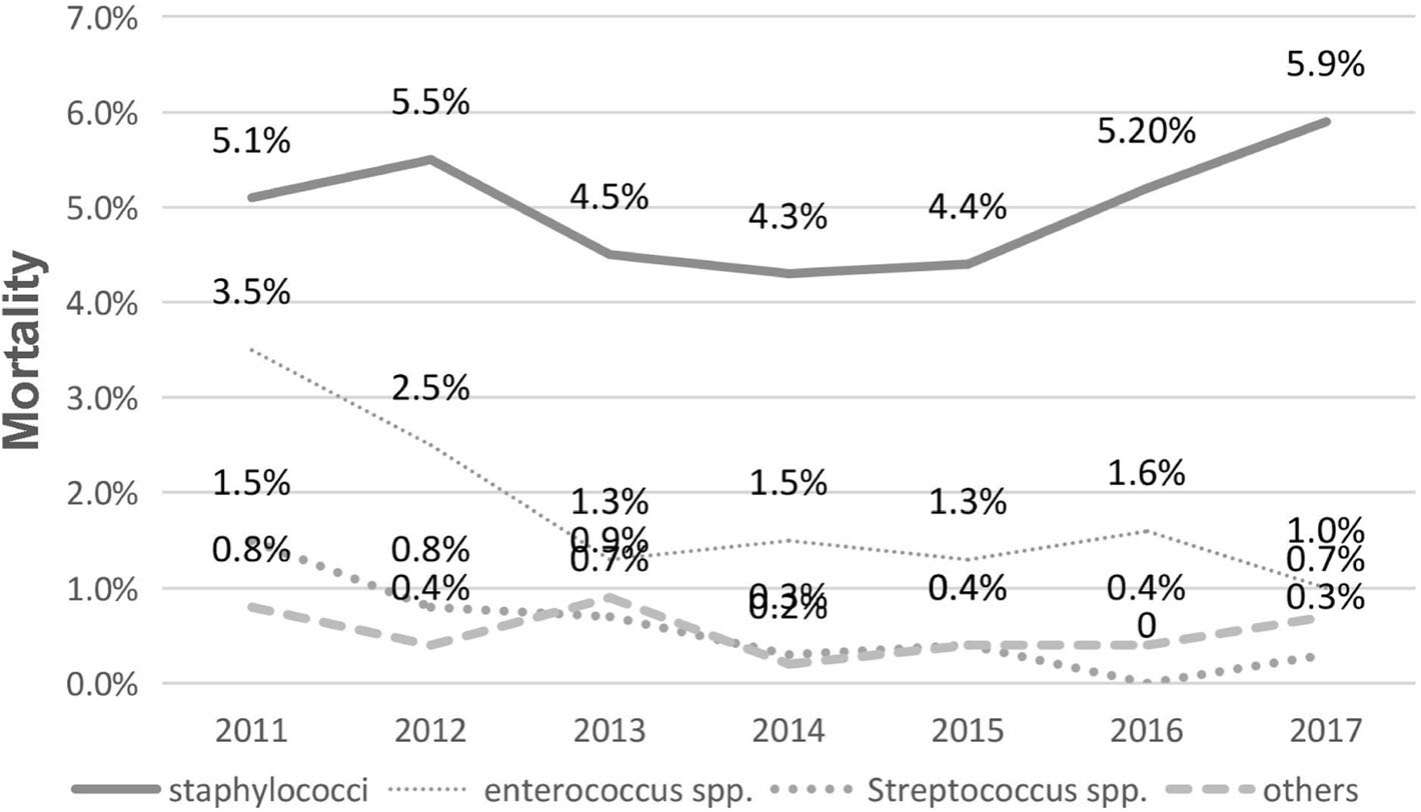
Mortality of the major gram-positive bacterials BSIs from 2011 to 2017(6-months’ data). The main pathogen causing death was *Staphylococci* (61.9%), followed by *Enterococcus spp.* (22.2%), *Streptococcus spp.* (7.6%) and others (6.3%). The mortality from *Staphylococci* (X^2^ = 0.81,*p* = 0.991) shows an increasing trend, while the mortality from *Enterococcus spp.*(X^2^ = 4.0,*p* = 0.677) and *Streptococcus spp.*(X^2^ = 14.62,*p* = 0.023) shows a downward trend

The mortality of Gram-positive bacterial bloodstream infections showed a downward trend from 2011 to 2017 (Fig. 2); The main pathogen causing death was *Staphylococci* (61.9%), followed by *Enterococcus spp.* (22.2%), *Streptococcus spp.* (7.6%) and others (6.3%). Mortality in patients infected with *Staphylococci* (X^2^ = 0.81, *p* = 0.991) showed an increasing trend, while the mortality in patients infected with *Streptococcus spp.* (X^2^ = 14.62, *p* = 0.023) and *Enterococcus spp.* (X^2^ = 4.0, *p* = 0.677) showed a downward trend (Fig. 3). Between 2011 and 2017, the mortality in the ICU decreased from 60.46 to 47.82%, and that in the general ward increased from 39.54 to 52.18%.

## Discussion

This study examined the incidence and characteristics of Gram-positive bloodstream infections in one of the largest tertiary-care hospitals in China. So far many studies have reported epidemiology, species distribution and antibiotic resistance of *Staphylococci*, *Enterococcus spp. Streptococcus spp.* Gram-positive bacilli and others [18–20], neverthless, only few studies have focused on Gram-positive bloodstream infections [21].

In our 6-year study, the incidence of Gram-positive BSIs switched order from 4.6 to 7.3 episodes per 10,00 admissions over 6 years. Acoording to our knowledge, thus far no such data, i.e. incidence rates of Gram-positive BSIs based on large retrospective studies have been reported. In the present study, a downward trend was observed in overall incidence in the ICU per year, while there was no obvious change on general ward. This might be explained with the increased importance of prevention and control infection among medical staff of ICU that was obvserved over recent years. Nevertheless, two recent studies haved reported an increase in the incidence of Gram-positive BSIs in general ward and ICUs [22, 23].

BSIs are commonly associated with comorbidities, such as malignancies, diabetes mellitus and infections [24, 6]. In the present study, we found that malignancy was the most common comorbidity, and the major predisposing factor for BSIs was indwelling central intravenous catheters; this data was consistent with previous results [25, 26]. Several studies have reported that the second most common factors is abdominal and lower respiratory tract infections [27, 18]; while we found that chemoradiotherapy was the second most common factor for BSIs. The observed differences might be due to the fact that the malignancy was the most common comorbidity with extensive use of intravascular catheters for chemotherapy and the high rate of radiotherapy, which more commonly lead to BSIs.

In our study, 69% of infections were healthcare-associated while 31% were community-associated (69% VS 31%). We hypothesized that this occurred due to the following reasons: first, the study population was mainly focused on healthcare-associated infection, and blood collection and culturing was a routine examination for hospitalized patients with fever. Another reason was that the common predisposing factors of Gram-positive BSIs such as central intravenous catheters, immunosuppression, and chemoradiotherapy were mainly found with hospitalized patients in our study. Beyond that, we have found that a rate of Streptococcus pneumoniae infection in China is significantly lower compared to other developed countries [28]. Pneumococcal vaccine is routinely used to treat *streptococcus pneumonia* disease in developed countries, which might affect the morbidity and mortality [29]. Nevertheless, China is still facing great challenges, such as uncomplete network for monitoring infection with streptococcus pneumoniae and lack of relevant immune policy for standardized use of pneumococcal vaccine. Moreover, besides PPV23 there is no available vaccine in China.

In our study, *Staphylococci* was 100% sensitive to vancomycin and linezolid except for *Staphylococcus epidermidis,* for which the vancomycin resistance rate was 0.13%. These results were consistent with the studies by Fayez et al. [30]. However, Mamishi and colleagues have reported an obviously higher prevalence of resistance to penicillin by *Streptococcus pneumonia,* i.e. 42.86% in Asian countries [31]. The reason might be the wide use of the variety of interventional procedures, immunosuppressive agents and vancomycin, so it is necessary to strengthen monitoring drug resistance and rational use of antibiotics in clinical. All of the cases of *Staphylococci* showed more than 90% resistance to penicillin except for MRSA. However, Vasudeva has reported that all *Staphylococci* showed are 100% resistant to penicillin [32, 33]. This may be related to the different practical uses of β-lactam antibiotics in various areas, resulting in different drug resistance to bacteria. Mover, the resistance rates of *Staphylococci* to gentamicin and tetracycline were less than 50%, *Staphylococcus epidermis* which was consistent with the previous studies [21, 34]. Therefore, both gentamicin and tetracycline might be good treatment choice against *Staphylococci* used to decrease the overwhelmingly dependent on vancomycin and linezolid.

In our study, all of the vancomycin-resistant isolates were *E. faecium* (4.1%), which was lower compared to number of other studies from US and Europe [35, 36]. Both *E. faecalis* and *E. faecium* have shown resistance to linezolid (3.8% and 3.1%), which was similar to the results from two studies from China [18, 21], nonetheless, resistance to linezolid was rarely reported in foreign studies [37]. This may be explained with the species distribution and antimicrobial resistance that varies geographically. In addition, there were 219 (44%, 95%CI 38–50%) patients who were treated with effective antibiotics before obtaining the report of antibiotic susceptibility. Test reports have shown that patients with *E. faecalis* BSIs are more likely to receive effective treatment by the empirical use of antibiotics (61% vs 35%, *p* = 0.013). This may be explained by the low resistance rate of *E. faecalis* to many antimicrobial agents, such as penicillin (9.1%), ampicillin (11.4%), gentamicin (31.4%), and ciprofloxacin (35.6%).

In our study, there was no vancomycin and linezolid resistance to *streptococcus pneumoniae* and *viridians streptococci*, which was consistent with the studies done by Fayez and Marshall [30, 33]. *Viridians streptococci* was 100% sensitive to penicillin, but two other studies have reported that penicillin resistance rates have increased to more than 50% [38, 39]. The penicillin resistance rate of *Streptococcus pneumoniae* was 30%, similar to many European countries (20% to 50%), although much higher rates were reported in other Asian countries [40, 41], while there were only a few countries with lower rates (less than 10%) [9, 32, 42]. Therefore, the penicillin aslo resulted as a treatment choice for *Streptococcus spp.*

In our study, the crude mortality of Gram-positive BSIs and the mortality of *Enterococcus spp.* and *Streptococcus spp.* have shown a significant downward trend from 2011 to 2017. One possible reason might be that the number, correct timing and accuracy of blood cultures were all greater in a tertiary-care hospital thus medical workers could more correctly choose antibiotics in a timely manner. Further, there is a greater choice of antibiotics in non-tertiary hospitals. Another reason might be that tertiary-care hospitals have more complete strategy for strict control of infection that can reduce the mortality factors in BSIs. The mortality in the ICU has shown a decreased trend, while the general ward revealed contrary results. This could be explained by certain proportion of surgical and critically ill patients at our hospital, although many previous studies have shown that these patients have higher mortality rates than patients at the general ward [22, 23]. Nonetheless, the diagnosis and treatment technologies of ICU such as ventilator and ECMO have been rapidly developing rapidly over recent 5 years, and a variety of positive treatment measures have shown effective to improve cure rate and reduce mortality. Some other reasons that highlighted the importance of infection control measures among the medical staff of ICU are strict aseptic operations, good hand hygiene and similar. On the contrary, the use of indwelling central intravenous catheter seem to be increasing because of more chemotherapy and venous nutrition employed at general ward, while the consciousness of medical staff related to BSIs remained weak and the maintenance measures related to central intravenous catheter remained not enough strict. Aslo and the patients at general ward later received the empirical treatment thus further increasing the mortality.

There are several limitations in the present study that should be considered. First, the collection of clinical data depended on medical records rather than interviews and the comprehensive assessment of clinical symptoms. Second, there was an inevitable bias this was a single-centre study, and some of the results might have been affected by the small sample size. Finally, many drug susceptibility results could not be evaluated because of a lack of unified standards aslo and not all isolates underwent the same antimicrobial agent sensitivity tests, so many isolates lacked resistance comparisons with other isolates in the present study.

## Conclusions

Gram-positive BSIs are the major cause of high morbidity and mortality, especially in patients with chronic disease and predisposing factors. However, in this study we found a decrease in morbidity and mortality, while the mortality at the general ward revealed the opposite trend. Although the resistance rates to vancomycin and linezolid among the Gram-positive BSIs increased, these antibiotics are still considered the best treatment options for patients with Gram-positive BSIs.

## Abbreviations

BSIs: Gram-positive bacterial bloodstream infections
CHINET: China antimicrobial surveillance network
CLSI: The Clinical and Laboratory Standards Institute
ECOM: Extracorporeal membrane oxygenation
ICU: Intersive care unit
MRSA: Methicillin-resistant *staphylococcus aureus*
MSSA: Methicillin sensitive *staphyloccus aureus*
NCCLS: National Committee for Clinical Laboratory
PLAGH: Chinese People’s Liberation Army General Hospital
RT-NISS: The real-time nosocomial infection surveillance system
